# Synchronization and leadership in string quartet performance: a case study of auditory and visual cues

**DOI:** 10.3389/fpsyg.2014.00645

**Published:** 2014-06-23

**Authors:** Renee Timmers, Satoshi Endo, Adrian Bradbury, Alan M. Wing

**Affiliations:** ^1^Department of Music, University of SheffieldSheffield, UK; ^2^Institute for Information-Oriented Control, Technische Universität MünchenMunich, Germany; ^3^School of Psychology, University of BirminghamBirmingham, UK; ^4^Royal Academy of Music, University of LondonLondon, UK

**Keywords:** timing, synchronization, leadership, ensemble performance, motion, movement, tempo, cues

## Abstract

Temporal coordination between members of a string quartet was investigated across repeated performances of an excerpt of Haydn’s string quartet in G Major, Op. 77 No. 1. Cross-correlations between interbeat intervals of performances at different lags showed a unidirectional dependence of Viola on Violin I, and of Violin I on Cello. Bidirectional dependence was observed for the relationships between Violin II and Cello and Violin II and Viola. Own-reported dependencies after the performances reflected these measured dependencies more closely than dependencies of players reported by the other players, which instead showed more typical leader–follower patterns in which Violin I leads. On the other hand, primary leadership from Violin I was observed in an analysis of the bow speed characteristics preceding the first tone onset. The anticipatory movement of Violin I set the tempo of the excerpt. Taken together the results show a more complex and differentiated pattern of dependencies than expected from a traditional role division of leadership suggesting several avenues for further research.

## INTRODUCTION

Synchronization between ensemble members contributes in important ways to the quality of a musical ensemble performance and can be seen as one of their performance goals. The micro-scale timings of a performance are, however, highly variable, largely due to expressive interpretations but also due to noise in the sensory-motor system. Resulting local and global tempo variations make temporal coordination between performers challenging. Traditionally, timing research has used a tapping paradigm to understand how the central nervous system controls the timing of motor execution with respect to an external event (Repp and Su, 2013). In this paradigm, people synchronize their finger tapping with a metronome set at various tempos and researchers study how the asynchrony between the tapping and the metronome is minimized. In a variant of the basic paradigm, the tempo of the metronome is fixed but unpredictably changed in phase, leading temporarily to an increased asynchrony. Within a few taps, the tapping adapts to the new phase of the metronome and the asynchrony is minimized (e.g., Repp, 2001). When a person synchronizes his/her timing with an autonomous “self-correcting” metronome (Repp and Keller, 2008) or with another person (Konvalinka et al., 2010), the tapping may be corrected to one or the other autonomous timing source or it may converge at a point intermediate to the two sources. These solutions to redundancy in the timing correction may be characterized in terms of a “leader” and “follower” of ensemble performance. Previous studies of these processes in musical contexts include investigations of piano duos (Goebl and Palmer, 2009) and most recently string quartets (Wing et al., 2014).

In particular, Wing et al. (2014) extended the first-order phase correction model derived from tapping studies (Vorberg and Wing, 1996; Vorberg and Schulze, 2002) to describe the dynamics of ensemble synchronization in a natural string quartet performance. Using a nested phase correction model, variations of the asynchronies between pairs of performers are described in terms of the dynamic interaction of timing correction between pairs of performers. One of the model’s predictions is that the stability of an ensemble’s togetherness is directly related to their ability to keep the level of asynchrony corrections across performers constant *in total*. That is, if one performer adjusts the asynchrony hardly at all, others need to compensate for it. In this way, the functional dynamics of the ensemble can be captured, with the dependence between players allowing for a characterisation of leader–follower relationships between performers.

Investigations of group dynamics in chamber music ensembles have suggested the relevance of leadership as well as democracy for the successful operation of such groups (Murnighan and Conlon, 1991). Within string quartets, artistic leadership is often attributed to the first violin (Violin I), while other members may take up other roles, organizational or social, or may function as “deputy” leader (King, 2006). The second violin (Violin II) may seem to have the least significant role by primarily supporting the melody, it is nevertheless essential to the success of the group, a phenomenon known as “the paradox of the second violin” (Murnighan and Conlon, 1991). Although these dynamics between ensemble members were observed in social interaction during rehearsals and concerts and discussed in interviews, it is likely that social and musical coordination have a close relationship (cf. Davidson and Good, 2002), where the social may reflect, as well as influence, the musical coordination.

Even when an ensemble has a leader who provides a primary reference for temporal coordination, it is still likely that individuals distribute their attention, responding to and correcting for asynchronies with other members of the ensemble. The degree of allocation of attention to timing across performers is of particular interest (Keller, 2001) and may vary depending on such factors as the perceptual salience of the instrument or the similarity in musical function between performers. This is an area that needs further investigation. Evidence exists that a tendency to predict a partner’s tempo is more beneficial to dyadic synchronization than when one of the partners is leading (Noy et al., 2011; Pecenka and Keller, 2011). The case may be different, however, for larger ensembles. As Rasch (1979) demonstrated, larger ensembles need a clearly uniting point of reference, such as a conductor, for successful synchronization.

While the asynchrony between tone onsets is an important acoustic cue, in regular performance contexts visual cues such as arm, instrument, head, and torso movements are also available to facilitate synchronization in ensemble performance. They may, however, not be the primary means for temporal synchronization. For example, Williamon and Davidson (2002) found that while head bends and eye contact between pianists increased over rehearsals, they were not prominent in the first rehearsal. Furthermore, Goebl and Palmer (2009) found that players in a piano duo made use of movement cues for synchronization when auditory feedback was limited but not in cases of full auditory feedback. In their study, the auditory feedback from the performers was controlled so that the pianists could only hear their own performance, or only one of the two performers received full auditory feedback. When the auditory feedback was reduced in this way, the performers voluntarily synchronized their head movements and the person assigned to be the leader demonstrated more exaggerated finger movements (lifting the fingers). These findings indicate that visual information about movement can aid synchronization (although not necessarily so, see Keller and Appel, 2010), but may come into play only as a secondary cue in support of auditory feedback.

In the investigation of the role of body movements for temporal synchronization, it is important to note that whole body movements tend to have a lower periodicity than that of the tone onsets (see, e.g., Timmers et al., 2006; Goebl and Palmer, 2009), instead corresponding to the duration of the measure, half-measure, or even phrase. These slower periodicities may assist interpretative coordination and have been used to quantify the degree of leadership (positive driving force) of string quartet performers (Glowinski et al., 2012). However, for tone to tone synchronization, relevant visual cues are more likely to be found in faster movements related to the production of sounds, for example finger movements in pianists (as investigated by Goebl and Palmer, 2009) and bow movements in string players (as investigated by Moore and Chen, 2010).

The present study contributes to the growing literature on timing and synchronization in musical ensembles by investigating temporal coordination and the role of auditory and visual cues in string quartet performance. Correlational methods are used to measure temporal dependencies between performers in the context of natural string quartet performances. We estimate these dependencies between performers by investigating correlated adjustments in the auditory and visual domain, assuming auditory synchronization to rely on tone onset timing and visual synchronization to rely on bow movement cues directly preceding tone onsets. Measured patterns of dependency are compared with self-reported degrees of dependency between pairs of performers.

## MATERIALS AND METHODS

### PARTICIPANTS

A professional string quartet, consisting of two male and two female performers, participated in this study. The quartet^1^ had played together for 10 years at the time of the study.

### MATERIALS

**FIGURE 1:**
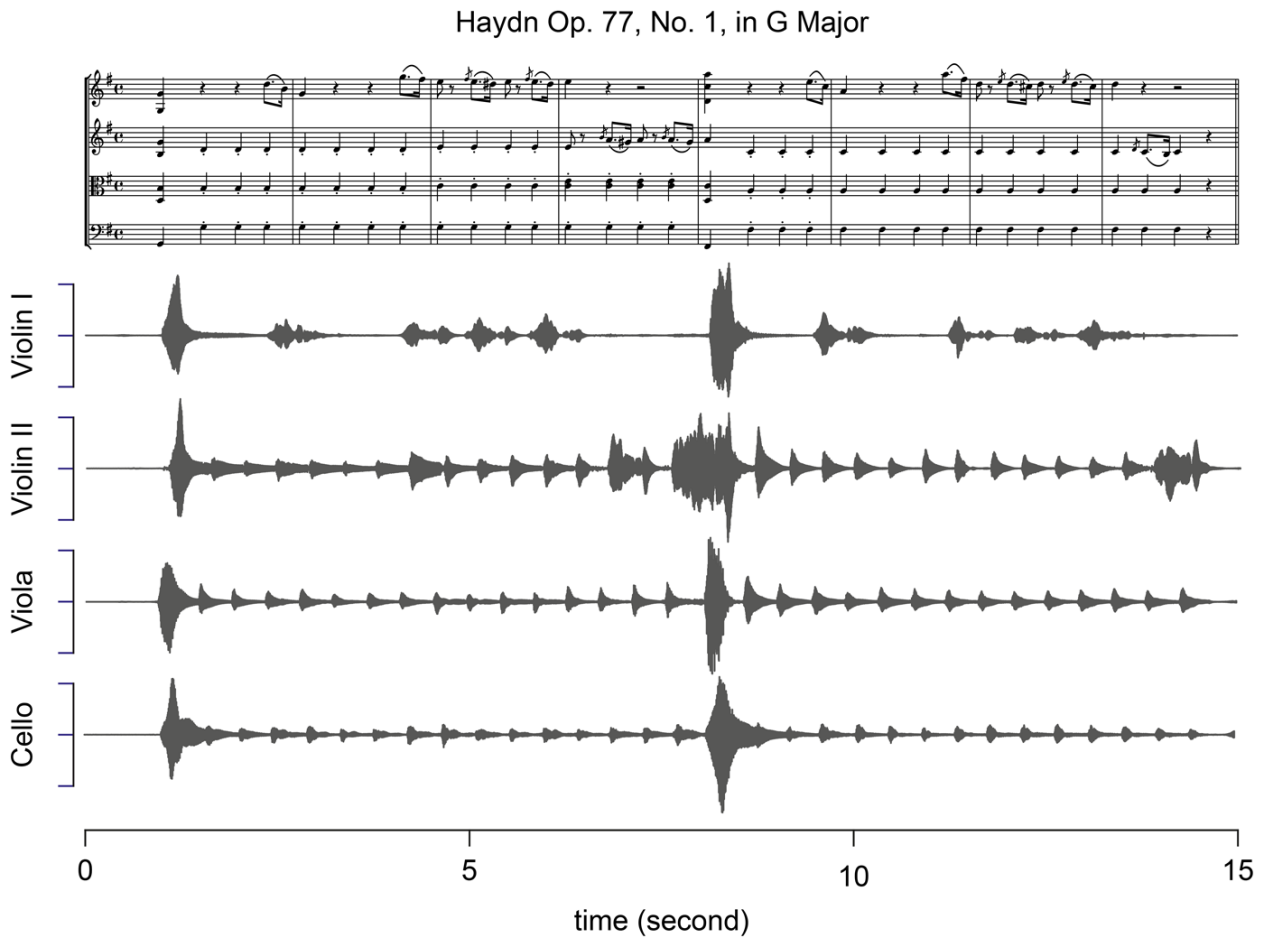
**The excerpt from the first movement of Haydn’s G Major string quartet, Op. 77 No. 1 played by the quartet and an example of audio data by each performer**.

The quartet performed the opening eight measures from the first movement of the string quartet in G Major by Joseph Haydn, Op. 77 No. 1 (see **Figure 1**). This excerpt was selected because of the relatively high proportion of synchronous tones across the two lower instruments. Violin I states a simple ornamented theme over four measures which is rhythmically repeated in the next four measures. The melody is echoed by Violin II, while Viola and Cello provide steady accompanying pulses throughout the eight measures.

### PROCEDURE

The quartet was seated in a circle of approximately 2 m diameter, in the sequence Violin I, Violin II, Viola, and Cello. They performed the musical excerpt 15 times, endeavoring to make each repeat an individual performance with some variation in interpretation. At the end of the block, participants indicated their subjective estimates of temporal dependencies between pairs of performers during the preceding performance, using a questionnaire: Firstly, the performers reported their dependence on each performer expressed as a percentage weighting assigned to each player including him/herself (the scores across the quartet summed to 100%). Secondly, they indicated the dependence that they expected each of the other performers would report.

The study was conducted in accordance with standard ethical procedures for research with human participants and ethical approval was obtained prior to the recordings. The performers gave their informed consent to participate in the study and for the researchers to use movement, video and audio recordings for analysis and demonstration purposes.

### DATA RECORDING AND ANALYSIS

Audio data were recorded using an omnidirectional miniature condenser microphone (Model 4061s, DPA Microphones A/S, Alleroed, Denmark) attached below the strings between bridge and tailpiece using a rubber clip (MHA6001, DPA). The microphone signals were sampled with a sound card (Model 8Pre, MOTU, MA, USA) at 44.1 kHz, and separately streamed and saved within Logic Studio Pro running on a MAC desktop PC (Apple, CA, USA). The audio data were formatted to uncompressed WAV files and analyzed in Matlab (MathWorks, MA, USA) off line. The audio data were rectified and then smoothed using a bi-directional second-order Butterworth low-pass filter with a cut-off frequency of 50 Hz (Bello et al., 2005). Local maxima of the signal corresponding to tones were detected, and tone onsets were determined using an adaptive threshold applied to the “valley” preceding each maximum. Only tones that coincided with quarter note beats were sampled, thus the grace notes and 16th notes of Violin I and II were avoided. This event detection method was visually cross-validated with their spectral analysis for the entire data set.

Kinematics of the performers were analyzed focusing on the speed of the bow with respect to the instrument. A 12-camera motion tracking system (Qualisys, Sweden) tracked the positions of retroreflective markers attached on the tip of the bow and the hand of each performer in 3D. Three more markers were attached to each instrument to provide a spatial reference for the bow and hand markers. The markers were attached to the bows and instruments using rubber bands, while double-sided tape was used to attach the marker to the hands of the performers. The sampling rate of the motion tracking was 200 Hz. The audio and motion tracking recordings were externally synchronized. The recorded kinematic data were smoothed using a Butterworth low-pass filter with a cut-off frequency of 12 Hz before further analysis. The speed of each bow movement was obtained by calculating the scalar absolute value of the velocity in 3D.

## RESULTS

### TEMPO VARIATION AND ASYNCHRONIES BETWEEN PERFORMERS

**FIGURE 2:**
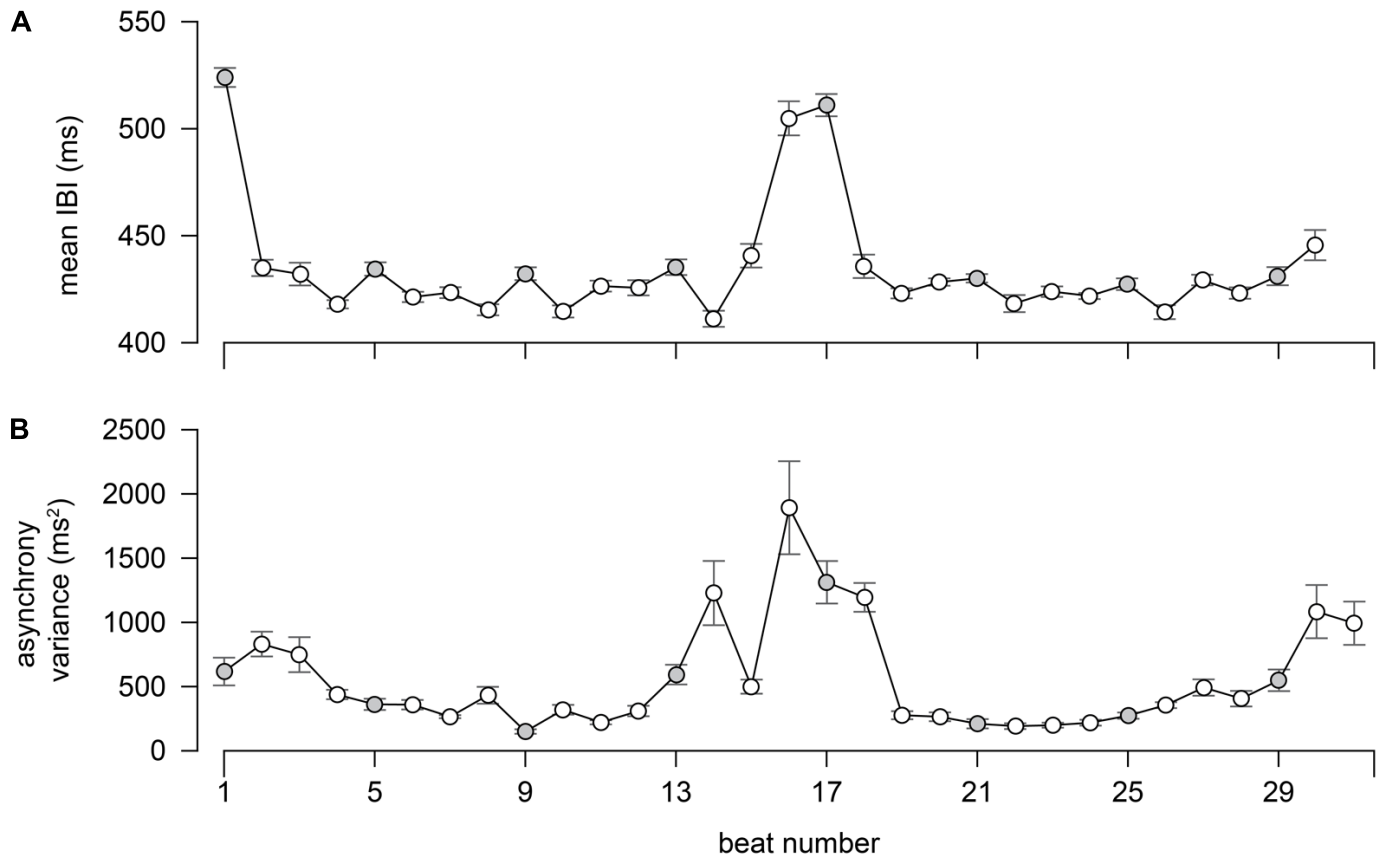
**Average IBIs (A) and asynchrony variances (B) of the quartet at each quarter note beat.** The error bars represent one standard deviation over repetitions.

**Figure 2** shows the variation in average interbeat interval^2^ (IBI) across the 31 quarter note beats of the excerpt as well as the asynchrony variances per beat. The IBI intervals were first averaged across the performers^1^ per beat. Then the average and standard error across trials of each IBI were calculated and reported in **Figure 2**. As can be seen in the figure, the opening chord is performed relatively slowly, as represented by a large IBI at the first interval (523.96 ± 17.16 ms). This is followed by faster and steady IBIs in the first phrase. At the 16th beat (final tone of the first four-measure phrase) and the 17th beat (start of the second four-measure phrase), the IBI increased to the same level as the entry IBI (504 ± 30.87 ms and 510 ± 20.18 ms, respectively). The inter-beat intervals then return to the faster and steady IBIs and the excerpt ends with a mild increase in IBIs (end of the eighth measure).

The asynchronies were calculated for each pair of performers, giving a total of 12 channels of asynchronies in a trial. The variances of these 12 sets of asynchronies were then calculated per beat and averaged across 15 trials to observe the structural characteristics of the asynchrony across the excerpt. As **Figure 2** illustrates, the changes in the asynchrony variance showed a similar pattern to the mean IBIs but were particularly large at the moments just preceding and following the phrase boundary at the 16th beat. In contrast, the variance at the first tone was considerably smaller than a correlation with the mean IBI at the first interval would suggest.

An analysis of the raw asynchrony values between pairs of performers revealed that the tone onsets of Violin I were consistently ahead of the other performers (Table 1). On the other hand, the onsets of Violin II were on average later than those of Violin I and Cello. The largest mean asynchrony was observed between Violin I and Viola, which was still very small (less than 15 ms on average) and the smallest mean asynchrony was observed between Violin II and Viola. These mean asynchronies suggest that Violin I and Cello take the lead in timing in contrast to Violin II and Viola who tend to follow. Cross-correlation analyses of IBIs at different lags will shed further light on this matter of leadership.

**Table 1:** Mean asynchrony for each pair of performers.

Asynchrony (ms)		Reference performer		
		Violin II	Viola	Cello
Comparison performer	Violin I	**-13.64 **(20.45)	**-14.99** (18.94)	**-**2.29 (13.89)
	Violin II		0.30 (8.63)	**12.41 **(9.63)
	Viola			**12.11** (8.18)

*Negative values indicate that the comparison performer is leading. SD are in brackets (*N *= 15). Asynchronies in bold are significantly different from 0 given the mean and SE with *t*-test values above 2.14 (*p* < 0.05).
*

### ESTIMATION OF DEPENDENCE BETWEEN PERFORMERS

Cross-correlation between IBI profiles of pairs of performers was used to assess the timing dependency between performers. It measures the association between the IBIs of performers at different lags, estimating the degree to which a performer makes similar variations in IBI at a subsequent or preceding instance (see Konvalinka et al., 2010). Cross-correlations were calculated after removal of changes in tempo as estimated from the average of the 15 repetitions, since these global tempo changes give a positive bias to the cross-correlations. Correlations were calculated at the beat level and per trial. In case of missing onsets due to rests in Violin I, the average onset time of the other voices was used as data point.

**FIGURE 3:**
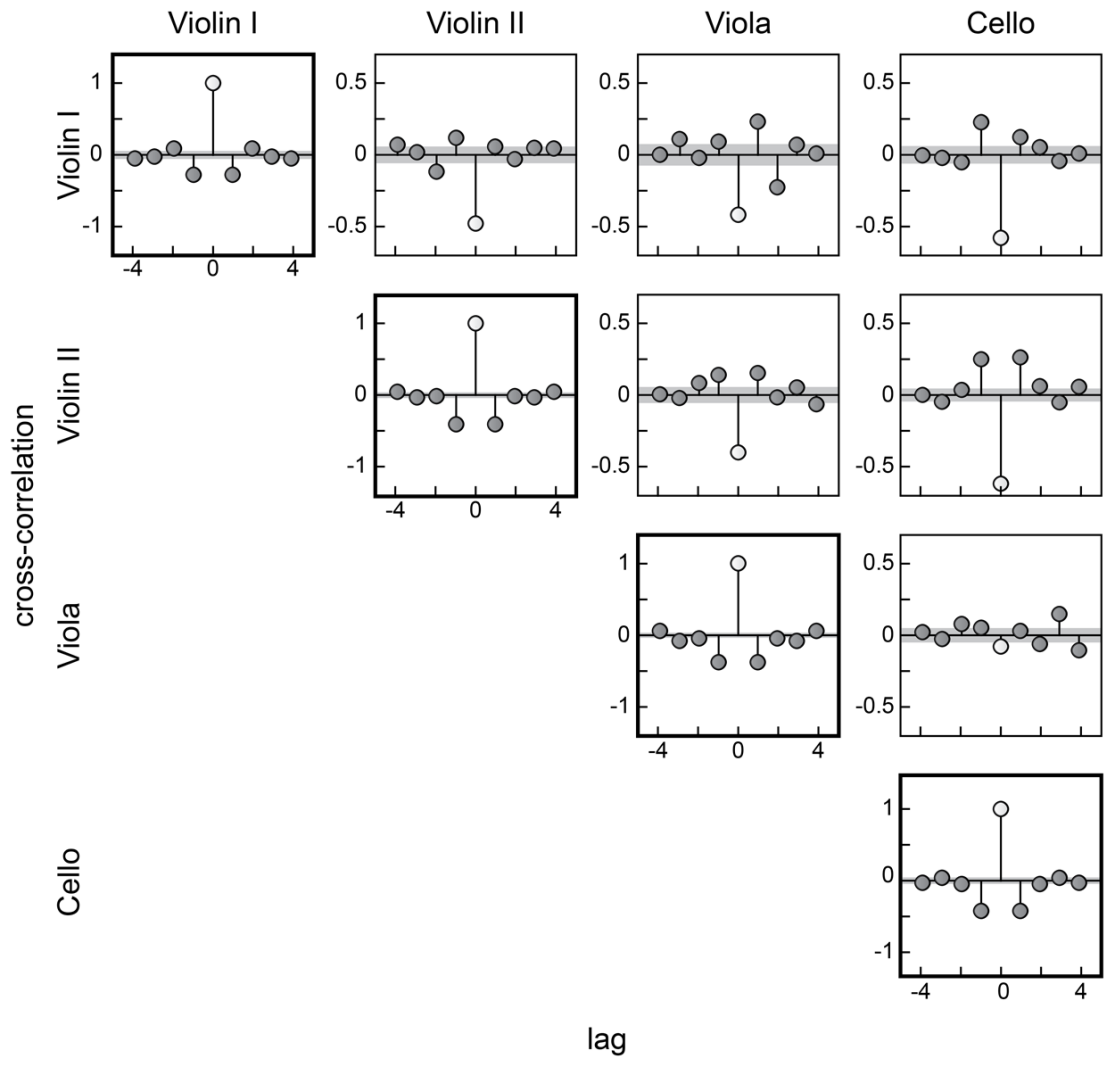
**A matrix of cross-correlation coefficients of IBIs between performers.** In the diagonal axis (bold squares), the autocorrelations are shown. Shaded areas indicate one standard error.

**Figure 3** shows an overview of the mean of between-instrument IBI correlations (averaged across trials). Grey areas indicate ± 1 SE. Auto-correlations are given along the diagonal. Within each box, correlations at different lags are given from a negative to a positive lag of four positions. In the case of negative lags, the voice in rows is shifted 1, 2, 3, or 4 beats backwards with respect to the voice in columns (the voice in rows is delayed). In the case of positive lags, the voice in rows is shifted forwards (the voice in rows is advanced).

Focusing on relatively strong positive cross-correlations indicating that one player followed the variations in IBI of another player, it appeared that Viola adapted to Violin I (first row, lag 1), but Violin I adapted to Cello. Relatively strong mutual adaptation was seen between Violin II and Cello, and weaker mutual adaptation between Violin II and Viola. These correlations are a multitude larger than the standard error, indicating their consistency across trials.

These results were supported by negative values at lag 0, which corroborate the idea of a first-order linear correction between performers. It indicates that performers were adjusting in opposite directions to maintain synchronization – where one performer compensated for a delay that he or she made by shortening an interval, the other performer responded by lengthening the interval in accordance with the delay. The coefficients were especially strong for correlations between Cello and Violin I, and Cello and Violin II, followed by other correlations with the violins. In the correlations between Violin I and Violin II, and Violin I and Viola, positive cross-correlations were coupled with a negative cross-correlation at a subsequent lag (-2 and 2, respectively). This may happen in cases of readjustment of the adjustment, resulting in opposite adjustments in subsequent lags.

Turning from cross-correlations to autocorrelations, negative correlations were observed at lag 1. This is consistent with the Wing and Kristofferson (1973) model of internal timing control where a lengthened (shortened) interval is followed by a shortened (lengthened) interval to maintain a steady tempo even without feedback correction. If feedback correction is used, this tends to further increase the negative lag 1 autocorrelation. All performers showed a similar degree of negative autocorrelations at lag 1, indicating a clear adjustment of timing variations within the subsequent beat.

### SELF-REPORTED DEPENDENCE BETWEEN PERFORMERS

**FIGURE 4:**
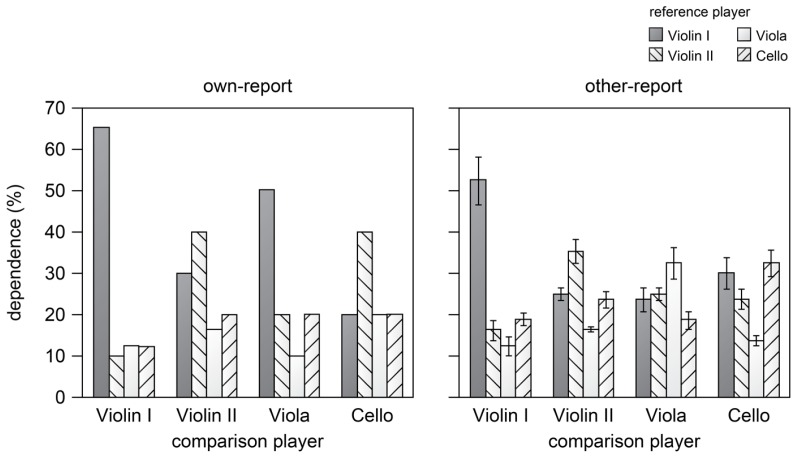
**Subjective ratings of the timing dependency of a performer (main player) on the timing of other performers (reference player) judged by the performer him or herself (left panel) and judged by the other performers (right panel).** The error bar represents one standard deviation.

The correlation-based measures of dependencies between performers were compared with self-reported dependencies. **Figure 4** shows the results of two types of self-reported dependencies between pairs of instruments. The left panel (“own”) shows the indications by performers of the extent to which their own timing (comparison player) depends on the timing of others or on themselves (reference player). The right panel (“other”) shows the mean ratings of the timing dependency of performers other than themselves, indicating how a particular player (comparison player) depends on other players (reference player).

The “own” results (left) show that ratings of dependence were highest for self in the case of Violin I and II. Viola indicated particular dependence on Violin I, and Cello relied on Violin II. This is in line with the measured dependencies, which also showed that Cello followed Violin II, despite the average negative asynchrony between the instruments (Table 1) showing Cello to be early. Violin I indicated to hardly depend on the others, which is in contrast to the measured dependencies, which indicated adaptation to Violin II and to Cello.

The “other” results (right) show the ratings of dependence of players judged by players other than the player him/herself. The ratings follow a consistent pattern in which highest estimates were given for dependence on “self” followed by dependence on Violin I and least dependence on Viola. The interrater reliability in terms of Pearson’s coefficient was reasonably high (*r* = 0.63 ± 0.163). These other-report patterns show less of the instrument specific patterns than the own-report and measured dependencies. For example, the dependence of Cello on Violin II was reported by Cello but not as strong by the others and Viola was reported by others to depend slightly more on Violin II than on Violin I, but indicated him/herself to depend primarily on Violin I, which is in line with the data.

### ROLE OF VISUAL CUES IN SYNCHRONIZATION

**FIGURE 5:**
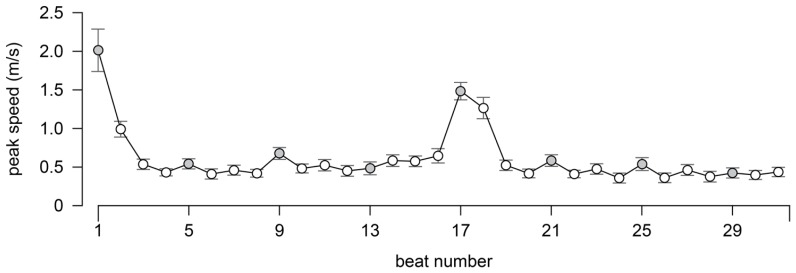
**The average peak speed of the bow movement directly proceeding to the acoustic onset.** The error bars represent one standard deviation.

As argued in Wing et al. (2014), ensemble timing is a result of players’ corrections for perceived asynchronies in acoustic properties such as tone onset or peak rate of change of spectral flux. Such corrections relate to the general pattern of IBI correlations described above. Whereas in the middle of a phrase, reliance on auditory cues may be sufficient, reliance on visual cues is necessary at the start of a phrase or the start of a movement. **Figure 5** depicts the peak speed of bow movements directly preceding the acoustic tone onsets. The variation in bow speed correlates with the average IBIs shown in **Figure 2**, indicating that bow speed was faster when preparing for longer as compared to shorter IBIs, which could be related to a shared underlying goal to emphasize particular notes in intensity and duration, where the increase in intensity is accomplished using higher bow speed.

The correlation between the peak speed and corresponding IBIs was consistently high for all performers. The correlation between the peak speed and the quartet average IBI was *r* = 0.67 (0.11) for the Violin I, *r* = 0.59 (0.10) for Violin II, *r* = 0.62 (0.11) for Viola and *r* = 0.67 (0.08) for Cello. One clear difference between the profiles is, however, that while IBIs lengthen at the end of the first phrase, the peak speed only grows at the start of the next phrase at beat 17 (where Violin I rejoins the ensemble after three beats of rest). Again, this is likely to be related to intensity where the performers lower the dynamics at the end of the phrase and start the next phrase with an accented downbeat in measure 5. The next analysis examines whether the peaks at the start of the phrase also function as a temporal cue.

**FIGURE 6:**
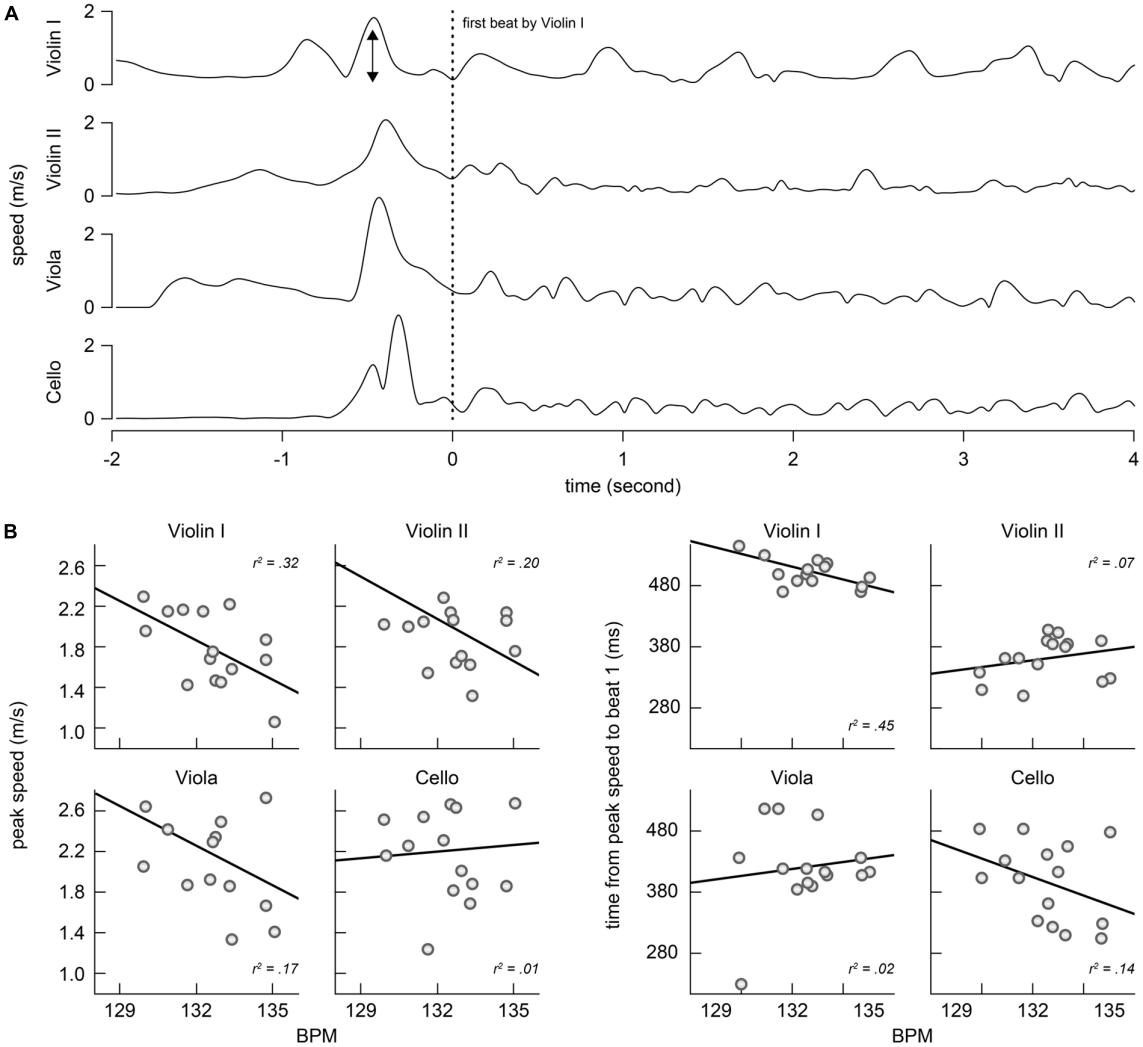
**(A)** Bow speed around the time of the first tone and **(B)** correlations between the overall tempo of the music (beat per minute) and peak profiles (peak speed and the time between the peak speed and the first tone onset).

A possible role for bow movement at points of entry is to indicate the tempo that is to follow. The top panel of **Figure 6** shows the average bow speed across trials for each instrument preceding and directly following the first tone onset. An arrow indicates the detected peak in bow speed for Violin I. This figure shows a double peak in bow speed of Violin I (at *t* = -0.9 s) that precedes the peak in bow speeds of the other instruments (at *t* = -0.5 s), suggesting that Violin I takes the lead. The bottom panel of **Figure 6** show the results of two analyses to investigate the hypothesis that the peak in bow speed is used as a temporal cue. The left panel shows the linear relationship between the average tempo of the excerpt and the peak speed of the bow movement preceding the first tone onset. The right panel shows the linear relationship between average tempo and the time between the peak speed and the onset of the first tone. Explained variances are given for each analysis and performer.

The speed profile of the upbeat movement by Violin I before stroking the first tone was indeed moderately correlated with the tempo of the music, which was observed in a reliable association between tempo and peak speed for Violin I (panel (**B**), left, *r*^2^ = 0.32) and between tempo and the time interval from peak speed and tone onset (panel (**B**), right, *r*^2^ = 0.45). This suggests that the bow movement prior to the performance provide a useful cue for grounding the tempo of the performance. Corresponding correlations with tempo were weaker for the other performers.

## DISCUSSION AND CONCLUSION

In this study, we investigated the temporal coordination between members of a string quartet in terms of measured cross-correlations in IBIs at different lags and in terms of self-reported dependencies. Additionally, we explored a possible role of visual cues for synchronization. The synchronization between the ensemble performers was high across the excerpt apart from the transition to the second phrase where the asynchrony variance peaked locally. Asynchrony variance was lower at the start of the excerpt than might be expected based on the absence of auditory cues for synchronization and the relatively slower tempo at the start of the movement. Analysis of bow speed highlighted that at the opening of the excerpt, Violin I seems to indicate the tempo of the excerpt through adjustments of the speed of the bow and the timing of the bow speed movement, using visual cues as tempo marking in the absence of auditory cues for synchronization. A special role of bow speed as visual cue at the starts of phrases was further suggested by relatively high speeds at the start and middle of the excerpt, although this may also be related to marking the starts of the phrases with greater intensity. The interpretation of the relevance of bow movement at the starts of phrases is corroborated by results reported in Wing et al. (2013) with the same musical excerpt, but a different quartet. Wing et al. (2013) compared the contribution of the movement of the head, left arm and right arm of Violin I to quartet synchronization. They asked Violin II, Viola, and Cello to play along with a complete or incomplete skeleton avatar constructed from the markers’ motion of Violin I. Absence of head markers or bow arm markers in the avatar visualization increased asynchrony in the quartet more than absence of the left arm markers. Increased asychrony was in particular present at the start and middle of the excerpt.

As highlighted by the cross-correlations between IBIs, synchronization during the performance was realized through a complex pattern of unidirectional and bidirectional dependencies between performers. Bidirectional adaptation was observed between Violin II and Cello, and between Violin II and Viola, while unidirectional patterns indicated that Viola was following Violin I, and Violin I was following Cello. These patterns of dependencies at the beat level are different from earlier reported dependencies at the measure level (see Timmers et al., 2013), which for example showed mutual dependence between Violin I and Cello. These variations complicate research, but are likely to be part of the complex reality of timing processes. The robustness of the results is indicated by the small variation of the measured correlations across repeated performances.

Patterns of dependencies were more closely reflected in the own-reported dependencies than in the other-reported dependencies. While the other-reported dependencies showed a pattern in which Violin I was leading and Viola was least depended on, the own-reported dependencies showed a more idiosyncratic pattern different for each performer. In line with the measured cross-correlations, Viola relied in particular on Violin I, Cello relied on Violin II, and Violin II relied on Violin I. Not indicated in the reported dependencies were the mutual dependencies between Violin II and Cello and between Violin II and Viola.

Interestingly, the mean asynchronies in tone onsets showed a different pattern of “leading” and “following” than that indicated by the cross-correlation analyses at different lags. The mean asynchronies indicated Violin I and Cello to be relatively early on average, while Viola and Violin II were on average relatively late. This reinforces a distinction between “leading” in terms of relative onset and “leading” in terms of setting the tempo or functioning as a temporal reference. Indeed, tapping studies illustrate their independence: while tapping to a metronome, the taps tend to anticipate the metronome (negative mean asychrony). Nevertheless, the metronome sets the tempo and is adjusted to (Repp and Su, 2013). In string quartet performances, relatively early or late onset of voices may be used to adjust the perceptual salience of these voices giving prominence to Violin I and Cello. Keeping together in time may, however, require a more dynamic process in which performers adjust to each other in a multitude of ways.

While the current study was exploratory and was not designed to test particualr predictions, further research needs to be done to examine to what degree performers adjust their timing strategies if the musical roles of those performers shift (e.g., if the melodic line is given to the Viola, and Violin I and II play the accompaniment), or if performers are explicitly instructed to follow someone. Manipulating the musical structure may in particular be effective as indicated by the results of Goebl and Palmer (2009) for piano duos. They found that note ratio between players affected patterns of dependency more than assigned leadership did, at least when auditory feedback was available.

It will also be necessary for future research to examine the consistency of the observed patterns across performances of the excerpt on different occasions or across performances of different excerpts, verifying whether the observed dependencies typify the ensemble. The measured correlations were consistent across trials, which indicates consistency within a performance session. Another avenue includes the investigation of temporal adjustment patterns with increasing expertise of ensemble performers – do beginner performers show more limited adaptation to others or show adaptation only at longer or shorter time-spans? Indeed, adjustments at multiple metrical levels will need to be systematically examined to gain a more comprehensive understanding of the processes involved.

With this case study of string quartet performance, we hope to have demonstrated that methodologies developed for solo and extended to duo performances can be further developed to investigate timing processes in larger ensembles of quartets, so contributing to the growing literature on ensemble performance. It seems that there is a limit to the number of co-performers to which performers adjust (and attention is payed to as also argued by, e.g., Keller, 2001). Indeed, we did not find any cases in which a performer adjusted to all other performers. This does not mean that attention is dedicated primarily to a single leader such as Violin I, nor that players have a single dominant reference player. While Violin I may lead at particular moments in the music such as the start, at other moments in the music, players’ adaptations to each other are more dynamic and mutual, compromising the notion of leadership while reinforcing the notion of the string quartet as a self-managed or self-organizing team (Gilboa and Tal-Shmotkin, 2012).

## Conflict of Interest Statement

The authors declare that the research was conducted in the absence of any commercial or financial relationships that could be construed as a potential conflict of interest.

## Footnotes

1 Quartet B in Wing et al. (2014).

2 Here we talk about interbeat interval rather than interonset or intertone intervals, because not all tones were included in the analyses – only the tones that coincided with a quarter note beat.

3 Average was calculated across three performers in case of rests in Violin I, otherwise across four performers.
